# Bioassay-Guided Chemical Study of the Anti-Inflammatory Effect of *Senna villosa* (Miller) H.S. Irwin & Barneby (Leguminosae) in TPA-Induced Ear Edema

**DOI:** 10.3390/molecules190710261

**Published:** 2014-07-15

**Authors:** Ana del Carmen Susunaga-Notario, Salud Pérez-Gutiérrez, Miguel Ángel Zavala-Sánchez, Julio Cesar Almanza-Pérez, Atilano Gutiérrez-Carrillo, Daniel Arrieta-Báez, Ana Laura López-López, Rubén Román-Ramos, José Luis Eduardo Flores-Sáenz, Francisco Javier Alarcón-Aguilar

**Affiliations:** 1 Doctorado en Biología Experimental, D.C.B.S., Universidad Autónoma Metropolitana, Unidad Iztapalapa, Av. San Rafael Atlixco No.186 Colonia, Vicentina, Iztapalapa 09340, Mexico; E-Mail: dioses_aztecas@yahoo.com.mx; 2 Laboratory of Productos Naturales, Departamento Sistemas Biológicos, D.C.B.S., Universidad Autónoma Metropolitana Unidad Xochimilco, Calzada del Hueso 1100, Col, Villa Quietud, Coyoacán 04690, Mexico; E-Mails: msperez@correo.xoc.uam.mx (S.P.-G.); mzavala@correo.xoc.uam.mx (M.Á.Z.-S.); 3 Laboratory of Farmacología, Departamento Ciencias de la Salud, D.C.B.S., Universidad Autónoma Metropolitana Unidad Iztapalapa, Av. San Rafael Atlixco No.186 Colonia, Vicentina, Iztapalapa 09340, Mexico; E-Mails: jcap@xanum.uam.mx (J.C.A.-P.); rrr@xanum.uam.mx (R.R.-R.); csib@xanum.uam.mx (J.L.E.F.-S.); 4 Laboratory of RMN, Departamento de Química, D.C.B.I. Universidad Autónoma Metropolitana-Iztapalapa, Av. San Rafael Atlixco No. 186, México D.F. 09340, Mexico; E-Mail: agrmn@xanum.uam.mx; 5 Instituto Politécnico Nacional-CNMN, Calle, Luis Enrique Erro s/n, Unidad Profesional Adolfo López Mateos, Gustavo A, Madero 07738, Mexico; E-Mail: danielarrieta@hotmail.com

**Keywords:** *Senna villosa*, anti-inflammatory effect, anti-proliferative properties, bioassay-guided chemical study

## Abstract

*Senna villosa* (Miller) is a plant that grows in México. In traditional Mexican medicine, it is used topically to treat skin infections, pustules and eruptions and to heal wounds by scar formation. However, studies of its potential anti-inflammatory effects have not been performed. The aim of the present study was to determine the anti-inflammatory effect of extracts from the leaves of *Senna villosa* and to perform a bioassay-guided chemical study of the extract with major activity in a model of ear edema induced by 12-O-tetradecanoylphorbol 13-acetate (TPA). The results reveal that the chloroform extract from *Senna villosa* leaves has anti-inflammatory and anti-proliferative properties. Nine fractions were obtained from the bioassay-guided chemical study, including a white precipitate from fractions 2 and 3. Although none of the nine fractions presented anti-inflammatory activity, the white precipitate exhibited pharmacological activity. It was chemically characterized using mass spectrometry and infrared and nuclear magnetic resonance spectroscopy, resulting in a mixture of three aliphatic esters, which were identified as the principal constituents: hexyl tetradecanoate (C_20_H_40_O_2_), heptyl tetradecanoate (C_21_H_42_O_2_) and octyl tetradecanoate (C_22_H_44_O_2_). This research provides, for the first time, evidence of the anti-inflammatory and anti-proliferative properties of compounds isolated from *Senna villosa*.

## 1. Introduction

The prevalence of atopic dermatitis has increased significantly, causing considerable economic costs and a decreased quality of life for its sufferers [1,2,3]. Different therapies are used in combination to treat atopic dermatitis to recover the functionality of the skin (hydrating creams), reduce itching (anti-histaminergic agents), prevent secondary infections (antibacterial agents) and control the inflammation process (steroidal and non-steroidal anti-inflammatories, NSAIDs/AINEs) [4]. The steroidal drugs can have severe side effects, such as constipation, sedation, respiratory depression and hallucinations, which may be annoying or dangerous for the patient [5]. These agents can also disrupt the release of various cytokines (TNF-α, IL-α y β, IL-2, IL-22) involved in leukocyte function, causing immunosuppression and, with chronic use, a decrease in the synthesis of collagen, producing epidermal atrophy [6,7]. AINEs also have a disadvantage because their prolonged use can cause gastric ulcers, blocked platelet aggregation, inhibited uterine motility, prolonged gestation and inhibited renal function [5,8]. Therefore, the discovery of new anti-inflammatory agents is needed.

Currently, research into traditional medicinal plants is a promising source for the development of new pharmacological agents. The genus *Senna* is comprised of approximately 350 species, mainly as a result of the new nomenclatural combination of non-American taxa [9]. Of these, 80% occur on the American continent, while most of the remaining members are found in tropical Africa, Madagascar, and Australia, with only a few species in southeastern Asia and some on the Pacific Island [9,10].

Chemical studies indicate that *Senna* species contain flavonoids, phenols, alkaloids, chromones, lactones, stilbenes, triterpenes, quinones and anthraquinones; other compounds isolated from the leaves are polysaccharides, flavonoids, sterols, terpenes, anthocyanins and proanthocyanidins, catechins, epicatechin, a flavonol glycoside, fistulic acid, chrysophanol, ascorbic acid, quercetin, amino acids, and cardiac glycosides. Many of these compounds have been associated with diverse biological activities, for example, laxative, antimicrobial, antifungal, antioxidant, hepatoprotective, antigenotoxic, hypolipidemic, spasmogenic and antinociceptive, antiproliferative, immunostimulatory, hypotensive, purgative, antidiabetic, estrogenic and antiestrogenic, antiulcer, antihistaminic, anti-cancer, anti-inflammatory, anti-nociceptive, antipyretic, platelet aggregatory and prostaglandin synthase activity [11,12,13,14,15,16,17,18,19,20,21,22,23,24].

The study of the anti-inflammatory properties of natural products has gained popularity, aimed at finding new agents without the aforementioned side effects and producing synthetic derivatives with enhanced activity [5,25,26,27]. In Mexico, the ethno-botanical information indicates a great variety of medicinal plants that are used to treat inflammation and the symptoms related to an inflammatory process [28,29]. Particularly in the State of Yucatán, located in southern México, the use of these medicinal plants is a common practice.

**Figure 1:**
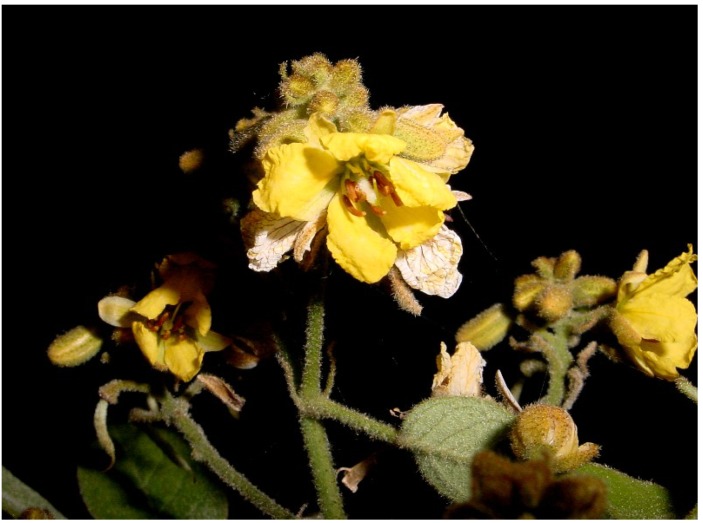
*Senna villosa*, popularly named saalche by the Mayan population.

*Senna villosa* (Miller) H.S. Irwin and Barneby (Syn*.*: *Cassia villosa* Miller, *Cassia articulate* Rose, *Cassia geniculata* Sesse and Mosino, *Cassia geniculata* Don, *Cassia stellata* Jones, *Chamaefistula astroites* Cham and Schidl) is a plant that grows in México in the states of Baja California, Campeche, Chiapas, Oaxaca, Puebla, Tabasco, Veracruz and Yucatán [30]. Popular Mayan names in Yucatán are salche, saalche, stall saalch’e, tsalche, tuy’ache, your ‘ja’ ché, boxsaal and zalche, whose meaning in Spanish is black bean (Figure 1) [31].

In traditional Mexican folk medicine, this plant is used topically to treat skin infections, pustules and eruptions and to heal wounds by scar formation. An aqueous extract is also used for the treatment of irregular menstruation and inflammatory problems [32]. It has been reported to have trypanocidal and antiprotozoal activities [33,34,35,36,37,38]. However, the anti-inflammatory activity of this plant and its active components has not yet been systematically studied.

The aim of the present study was to determine the anti-inflammatory effect of different extracts obtained from the leaves of *Senna villosa* in a model of ear edema induced by 12-*O*-tetradecanoylphorbol 13-acetate (TPA). The extract that presented the most activity was fractioned, and after isolation and purification, the chemical structure of the active compound was established.

## 2. Results and Discussion

### 2.1. Anti-Inflammatory Effect

Table 1 shows the data obtained from the evaluation of the anti-inflammatory activity of the *Senna villosa* extracts in the model of ear edema induced with TPA. The ears treated only with the vehicle exhibited a normal mean weight of 9.37 ± 0.29 mg and did not develop edema. The ears that received only TPA/vehicle (the control group) developed an edema of 10.23 ± 0.39 mg 6 h after the TPA application. The ears treated with indomethacin showed a significantly lower edema compared with the control group, in which edema was inhibited by 36.15% ± 4.75%. The group treated with the chloroform extract showed a percentage of inhibition of edema of 57.96% ± 5.21%, which was the most intense in relation to indomethacin (*p* < 0.0001).

**Table 1:** Anti-inflammatory effect of different extracts from *Senna villosa* leaves in TPA-induced ear edema.

Group	Topical Dose/Ear (mg)	Edema (mg)	% of Inhibition
TPA-Control	Vehicle	10.23 ± 0.39	-
Indomethacin	2	6.53 ± 0.48 **	36.15 ± 4.75
Chloroform Extract	2	4.30 ± 0.55 **^,^^♦^	57.96 ± 5.21 ^♦^
Methanol Extract	2	9.11 ± 0.29	10.90 ± 2.88
Aqueous extract	2	7.83 ± 0.59 *	23.42 ± 5.78

Mean ± S.E.M. ANOVA followed of Tukey’s multiple comparisons test. Significant differences compared with TPA-control (* *p* < 0.05, ** *p* < 0.0001; *n* = 10). Significant differences compared with indomethacin (^♦^ *p* < 0.0001; *n* = 10).

The methanol extract did not show anti-inflammatory activity, while the aqueous extract showed a percentage of inhibition of 23.42% ± 5.78%, which was minor in comparison to the values exhibited by the chloroform extract. Based on these results, the leukocyte differential count and the chromatographic separation were performed only on the chloroform extract.

The present study evaluated the anti-inflammatory effect produced by different *Senna villosa* leaf extracts (chloroform, methanol and aqueous) on TPA-induced edema, finding that the chloroform extract was the most active. Our results reveal that topical application of the chloroform extract inhibits edema in a model of acute inflammation in the mouse ear. It is worth mentioning that the chloroform extract of *Senna villosa* also inhibits the inflammation associated with allergic contact dermatitis in this mouse model, showing that this extract is effective against both innate and adaptive immune responses.

The epidermis is exposed to stimuli from both outside and inside the body, which affect cytokine production. Cytokines have an important impact on the body’s ability to react appropriately to its environment and to overcome systemic diseases. *In vivo* studies provide important clues about the possible role of cytokines in various skin diseases and also allow for the assessment of cytokines as possible therapeutics [39].

TPA-induced inflammation in the mouse ear has been routinely used to test whether topically applied anti-inflammatories inhibit the development of chemically induced acute dermal irritation [40,41,42,43,44,45,46,47] or adaptive immune responses, such as allergic contact dermatitis [48,49,50]. This model induces cutaneous inflammation in the ear for topical applications, which induces the cellular hyper-proliferation that is observed in diseases of the skin, such as psoriasis and atopic dermatitis [51]. To understand the inflammatory process induced by TPA, and thus establish the possible mechanism of action of the tested compounds, it is important to know the structure of the skin, including its composition of cell types and which signaling pathways are activated by this agent.

### 2.2. Leukocyte Differential Count

The results of the leukocyte differential count performed on blood smears from the animals treated with the chloroform extract are shown in Table 2. The time zero represents the mean number of quantified leukocytes before TPA application, which were similar to those reported by Harlan Laboratories for each cellular type and did not show significant differences between the different treatments (*p* > 0.05).

**Table 2:** Temporal curse of the leukocyte differential count in the model of TPA-induced ear edema.

Celular Type	Time	TPA-Control	Indomethacin	Chloroform Extract
**Neutrophil**	0	54.50 ± 9.75	37.53 ± 2.91	46.72 ± 6.21
	240	34.69 ± 4.13	40.11 ± 6.84	80.72 ± 1.63 * ^♦,●^
	300	38.77 ± 1.72	55.88 ± 6.99	66.32 ± 2.90 ^♦^
	360	91.15 ± 2.98 *	40.37 ± 2.21	81.04 ± 1.95 * ^♦,●^
**Monocyt**	0	3.17 ± 1.10	2.01 ± 0.24	1.50 ± 0.62
	240	1.23± 0.24	2.39 ± 0.60	1.75 ± 1.28
	300	1.23 ± 0.24	0.67 ± 0.37	5.75 ± 1.75 ^♦,●^
	360	1.65 ± 0.30	3.32 ± 0.33	8.99 ± 1.76 * ^♦,●^
**Lymphocyte**	0	54.98 ± 1.24	55.37 ± 3.39	51.72 ± 6.17
	240	50.93 ± 2.53	53.75 ± 6.99	18.13 ± 1.76 * ^♦,●^
	300	43.00 ± 1.61	38.88 ± 6.40	30.48 ± 3.30 *
	360	35.55 ± 4.25 *	54.80 ± 1.54 ^♦^	14.15 ± 2.04 * ^♦,●^
**Eosinophil**	0	0.33 ± 0.22	0.59 ± 0.07	0.58 ± 0.31
	240	1.06 ± 0.44	0.46 ± 0.14	0.12 ± 0.12
	300	1.60 ± 0.37 *	0.44 ± 0.21	0.37 ± 0.18
	360	2.21 ± 0.41 *	0.55 ± 0.12 ^♦^	0.12 ± 0.12 ^♦^
**Basophil**	0	0.65 ± 0.25	0.38 ± 0.21	0.27 ± 0.18
	240	63.02 ± 4.69 *	0.59 ± 0.26 ^♦^	0.12 ± 0.12 ^♦^
	300	58.45 ± 1.85 *	2.13 ± 1.89 ^♦^	0
	360	53.54 ± 2.45 *	0.10 ± 0.10 ^♦^	0

Mean ± S.E.M. ANOVA followed by the test of Tukey’s multiple comparisons. Significant differences compared with time zero (* *p* < 0.05; *n* = 10). Significant differences compared with TPA-Control (^♦^ *p* < 0.05; *n* = 10). Significant differences compared with indomethacin (^●^ *p* < 0.05; *n* = 10).

The TPA-control animals showed a significant decrease in lymphocytes and increase in eosinophils and basophils at 360 min. Compared with time zero, the animals treated with indomethacin after the administration of TPA did not show changes in their leukocyte count during the experiment. The animals administered the chloroform extract showed a significant increase in neutrophils and monocytes. In addition, significant reductions of lymphocytes, eosinophils and basophils were also observed (Table 2).

The skin does not only serve as a physical and chemical barrier but is also an immune-competent organ that elicits effective innate and adaptive immune responses to protect the body. Cells in the dermis and epidermis alike, including dermal dendritic cells, epidermal Langerhans cells, melanocytes, keratinocytes and migrating lymphocytes, are important in this process and are known to produce a great variety of cytokines [52]. Skin inflammation comprises a plethora of physiologic reactions, involving mainly the blood vessels and the subjacent connective tissue. This response is characterized by hyper-proliferation of the skin epidermal layer, which is attributed to a premature maturation of keratinocytes and dermal inflammatory infiltrates, comprising dendritic cells, macrophages and T-cells. Inflammation is maintained by the interactions of local cell types, such as nerve terminals, keratinocytes, fibroblasts, mast and endothelial cells and macrophages. The pronounced inflammation induced by topically administered TPA is mediated by protein kinase C and the stimulation of phospholipase A2 and cyclooxygenase. These events cause the release of arachidonic acid and prostaglandin E2 [43,53]. TPA induces changes in vascular permeability parallel to the increased edema, with local accumulation of neutrophils, monocytes and macrophages [54] and epidermal hyperplasia [55,56,57,58,59,60]. There are no reports of changes at the systemic level.

The growth and differentiation of epidermal keratinocytes have shown to be PKC-regulated [61,62]. Alternatively, keratinocyte-released mediators could affect G-protein-coupled receptor signaling in other dermal cells involved in neutrophil attraction and extravasation. In this model, some important mediators participate in the inflammatory reaction, and TPA seems to induce the expression of pro-inflammatory cytokines in keratinocytes [63,64]. These data were also confirmed *in vitro* because Murakama *et al.* [65] showed that the addition of TPA to human keratinocytes resulted in an increase in the amount of TNF-α.

Through the differential leukocyte count, it was possible to establish a systemic effect caused by the topical application of TPA to the ears of mice. In addition, this technique enabled correlation of the changes in the levels of different cell types to the inflammatory mediators involved in this experimental model. Often, however, cytokines released at the side of inflammation facilitate both the adherence of immune-system cells to vascular endothelial cells and their migration through the vessel wall into the tissue spaces. We see this reflected in the results obtained in the differential leukocyte count, the TPA-control animals showed a significant decrease in lymphocytes and increase in neutrophils, eosinophils and basophils at 360 min.

Many types de leucocytes move from one part of the body to another. This is especially true of lymphocytes, which circulate continually in the blood and lymph and, in common with other types of leukocytes, migrate into the tissues at tissues injury. Lymphocytes are capable of a remarkable level of recirculation, continually moving through the blood and lymph to the various lymphoid organs. This recirculation of lymphocytes would explain the decrease observed after administration of TPA. The injured site also attracts granulocytes (neutrophils, eosinophils and basophils) that migrate to the inflamed tissue. This explains the increase of these leukocytes; which in turn induce the release of inflammatory mediator cytokines and thus creating a complex feedback system.

The result is an influx of the lymphocytes, neutrophil, monocytes, eosinophils, basophils and mast cells to the site of tissue damage, where these cells participle in clearance of the antigen and healing of the tissue. Clearly, the processes of the leukocyte adhesion are of great importance in the inflammatory response. The behavior of these leukocytes allows us to understand and correlate other inflammatory mediators that are closely related.

**Figure 2:**
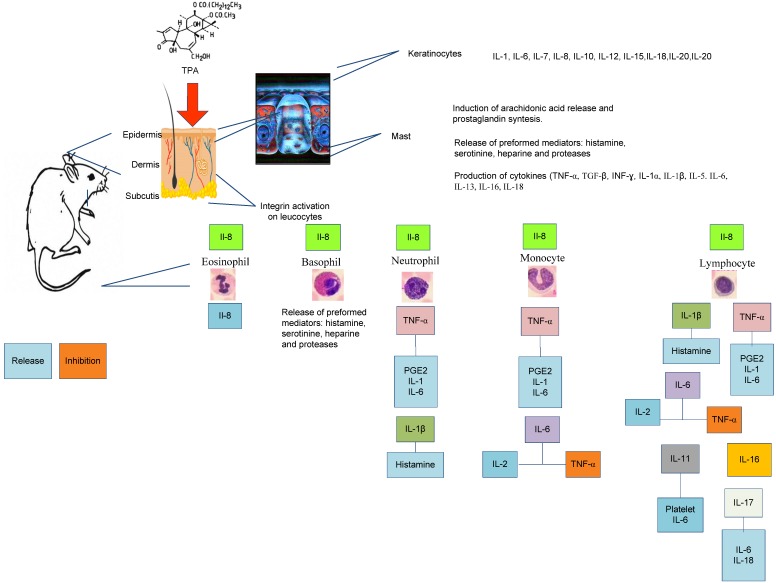
Inflammatory mediators after application of TPA.

These results permit the establishment of appropriate times for the quantification of systemic inflammatory mediators, such as histamine, serotonin, lysosome enzymes, prostaglandins, leukotrienes, platelet activating factor, reactive oxygen species, nitric oxide and cytokines, in a close relationship with changing blood leukocyte levels. Because the average numbers of the different cell types were observed until 6 h post-treatment, other biomarkers should be measured at this time point to elucidate the anti-inflammatory mechanism of action at the systemic and local levels. Of particular relevance, markers of oxidative stress (total nitric oxide) and the expression of the genes involved in TPA-induced inflammatory response should be considered. Therefore, quantification of cytokines in the atrial tissue (at the local level) and in the serum (systemic level) should be performed in future studies (IL-1, TNF-α, IL-6, IL-10 and IL-12, in addition to PGE2 and COX-2), between 240 and 360 min post-treatment to elucidate the mechanism of action of the principally active extracts of *Senna villosa* (Figure 2 describes this idea).

Given the structure and composition of the mice ear skin, the major cell types stimulated after application of TPA are keratinocytes and mast cells, however, many of the currently identified cytokines are produced by keratinocytes, either constitutively or upon induction by various stimuli. Such cytokine production by keratinocytes has multiple consequences for the migration of inflammatory cells, which may have systemic effects on the immune system, influences on the keratinocyte proliferation on the differentiation processes, and finally it affects the production of other cytokines by keratinocytes. The injured site also attracts granulocytes that migrate to the inflamed tissue [6]; which in turn induce the release of inflammatory mediators cytokines and thus creating a complex feedback system.

### 2.3. Fractionation of the Chloroform Extract

Based on the fact that the chloroform extract had the highest anti-inflammatory effect, it was chromatographically separated, yielding nine fractions. The fractions and weights obtained were as follows: 1 (0.063 g), 2 (0.075 g), 3 (0.1803 g), 4 (0.0116 g), 5 (0.0666 g), 6 (0.0972 g), 7 (0.5751 g), 8 (0.3659 g), 9 (0.1279 g). Fractions two and three rendered a white precipitate (0.1874 g).

### 2.4. Bioassay-Guided Chemical Study (Anti-Inflammatory Effect in the Model of Ear Edema Induced by TPA)

The ears that only received TPA and vehicle (TPA-control) developed an edema of 12.18 ± 0.68 mg after 6 h of TPA application (Table 3). The TPA-treated ears that were also treated with indomethacin (2 mg/ear) showed a significant reduction in edema of 43.90% ± 5.81% (weighing 6.83 ± 0.70 mg).

**Table 3:** Evaluation of the anti-inflammatory effect of the white precipitate from fractions 2 and 3 of *Senna villosa* leaves chloroform extract in TPA-induced ear edema.

Group	Dose topically Administered/Ear (mg)	Edema (mg)	% of Inhibition
Control	STPA-control	12.18 ± 0.68	-
Indomethacin	2	6.83 ± 0.70 *	43.90 ± 5.81
Fraction 1	2	11.46 ± 0.63	5.91 ± 5.42
Fraction 2	2	9.18 ± 0.79	24.63 ± 6.55
Fraction 3	2	10.10 ± 0.88	17.08 ± 7.29
Fraction 4	2	8.66 ± 0.58	28.90 ± 4.82
Fraction 5	2	10.16 ± 0.66	16.58 ± 5.45
Fraction 6	2	9.51 ± 0.91	24.88 ± 7.51
Fraction 7	2	10.05 ± 0.49	17.49 ± 4.05
Fraction 8	2	10.98 ± 0.47	9.82 ± 3.93
Fraction 9	2	11.13 ± 0.35	8.66 ± 2.94
White precipitate from fractions 2–3	2	6.30 ± 0.93 *	48.28 ± 7.64

Mean ± S.E.M. ANOVA followed by Tukey’s multiple comparisons test. Significant differences compared with control group (* *p* < 0.001; *n* = 10).

Although the nine fractions obtained from the chloroform extract did not show anti-inflammatory activity, the majority of the fractions exhibited tendencies to reduce the edema. From the nine fractions, fraction 4 exhibited the best tendency anti-inflammatory, with 28.90 ± 4.82 of percentage of inhibition of the edema; and in similar way to the other 8 fractions, the difference did not present statistical significance, compared with control (*p* > 0.05).

The chemical composition of the extracts of medicinal plants is very varied, and different chemical studies show the presence of more than one compound with various pharmacological properties of interest. The chloroform extract of *Senna villosa* contain several non-identified compounds that may have synergistic activity. In fact, the majority of the nine fractions obtained from *Senna villosa,* which might contain different kind of compounds, exhibited tendencies to reduce the edema. Fraction 4 exhibited the best tendency anti-inflammatory, with 28.90 ± 4.82 of percentage of inhibition of the edema; and in similar way to the other fractions showed certain activity. It is clear that administered all together in the chloroform extract, these fractions exhibit better activity than indomethacin and white precipitate. Therefore, our results suggest a probable synergistic effect among these different chemical compounds present in the chloroform extract of *Senna villosa.*

The white precipitate isolated from fractions two and three was active, showing a percentage of inhibition of edema of 48.28 ± 7.64 (weighing 6.30 ± 0.93 mg). The white precipitate was subjected to dose response testing. The tested doses were 0.5, 1 and 2 mg/ear (data not shown). All the doses showed inhibition of the edema (*p* < 0.05). The dose at which the best activity was observed was 2 mg/ear. For this reason, this dose was chosen to perform experiments with the white precipitate.

### 2.5. Structural Elucidation (NMR, IR, MS)

The ^13^C-NMR spectrum showed signals at 173.85 and 64.3 ppm that were assigned to a carbonyl carbon and to the α carbon of ester oxygen, respectively. Both of these signals confirmed the presence of ester moieties. The rest of the carbons were assigned by 2D spectra (COSY, HSQC and HMBC). The presence of the ester group in the proposed structures was evidenced by the ATR-FTIR spectrum. The bands at 1,729.6 cm^−1^ corresponded to the carbonyl stretching, while 1,171.7 belonged to the stretching of the C-O bond.

In the negative ion mode electrospray mass spectrum of the white precipitate three deprotonated molecular [M−H]^−^ ions (*m/z* values, 113.0289, 265.2494 and 325.3099) were apparent. The latter had the greatest proportion. The negative ion mode electrospray mass spectrum of the deprotonated molecular ion [M−H]^−^, with the *m/z* that made up the greatest proportion of the white precipitate. Table 4 shows the proposed structures for the deprotonated molecular ions [M−H]^−^, with *m/z* values of 311.2890, 339.3308 and 325.3099.

Previous phytochemical studies of *Senna villosa* leaves revealed the presence of quinones, sterols, flavonoids and tannins. In the hexane extract from the leaves, two anthracenic quinone-like main components were identified as chrysophanol and fiscion [32]. Three components were isolated from the dichloromethane extract: squalene (triterpene), chrysophanol and fiscion (anthraquinones). From an ethyl-acetate fraction, seven secondary metabolites were identified: emodin (anthracenic quinone), apigenin, luteolin and chrysoeriol (flavones), quercetin and kaempferol (flavonols) and demetoxicapilarisina (neoflavone) [66]. Analysis of the essential oil from the seeds revealed the presence of the ethyl esters of palmitic acid and stearic acid and the methyl esters of linoleic acid, stigmasterol, δ-sitosterol and vitamin E [67]; (8-hydroxymethylene)-treicosanyl acetate was isolated of the chloroform extract [37]. Several of these compounds have been associated with biological activity. For instance, anthraquinone glycosides isolated of various plants from *Senna* genus exhibited laxative activities. However, in Mexico there are no reports about a traditional use of *Senna villosa* as laxative.

**Table 4:** Molecular ion, molecular formula and molecular structure of the main components of the white precipitate from fractions 2 and 3 of the chloroform extract.

Molecular Ion	Chemical Formula	Structural Formula
311.2890	C_20_H_40_O_2_	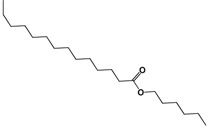
		1.-Hexyl tetradecanoate
325.3099	C_21_H_42_O_2_	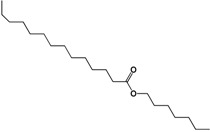
		2.-Heptyl tetradecanoate
339.3308	C_22_H_44_O_2_	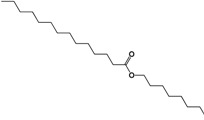
		3.-Octyl tetradecanoate

Three aliphatic esters were identified as principal components of the white precipitate from fractions 2 and 3 of the chloroform extract of the *Senna villosa* leaves, which had not yet been reported in this specie: hexyl tetradecanoate (C_20_H_40_O_2_), heptyl tetradecanoate (C_21_H_42_O_2_) and octyl tetradecanoate (C_22_H_44_O_2_). However, there have been several studies of these compounds obtained through synthesis or isolated from different sources. The only pharmacological activity of these compounds reported to date is antibacterial [68].

## 3. Experimental Section

### 3.1. Plant Material

*Senna villosa* was gathered in September of 2011 in the rural community of Komchem, 17 km from Merida, Yucatán, and was authenticated by Salvador Flores Guido at the Herbarium of the “Universidad Autónoma de Yucatán” (voucher number 10284). The biological material was air dried at ambient temperature for seven days, until a complete dehydration was achieved. The leaves of the plant were then ground by means of a manual stainless steel mill (ROTTER).

### 3.2. Preparation of Extracts

The dried leaves of *Senna villosa* (500 g) were consecutively refluxed for 4 h in 3.5 L of chloroform, methanol and water and then filtered. The organic solvents were eliminated at reduced pressure in a rotary evaporator (Buchi R-200), while the aqueous extract was freeze-dried (Labconco freeZone). The yields of chloroform, methanol and water extracts were 22.32%, 15.78% and 8.56%, respectively.

### 3.3. Animal Experiments

Male CD-1 mice, approximately six weeks old and 25–30 g, were used. The animals were provided by the animal center at Universidad Autónoma Metropolitana Unidad-Iztapalapa. They were raised at ambient temperature, with a 12:12 h light/dark cycle and allowed food (Harlan^®^, México D.F., México) and water *ad libitum.* The handling of the laboratory animals was performed in agreement with the statutes of the CICUAL (Institutional Committee for the Care and Use of the Animals) based in the international and national rules established in the “Official Mexican Rule” for the care and use of the laboratory animals” [NOM-062-ZOO-1999, revised in 2001) [69].

### 3.4. Anti-Inflammatory Effect in the Model of Ear Edema Induced by TPA

The animals were separated into five groups of ten animals. Edema was induced by TPA (Sigma-Aldrich, St. Louis, MO, USA), which was topically administered to the left ear of every mouse at a dose of 2.5 μg of TPA, dissolved in 25 μL of acetone. Thirty minutes after this TPA administration in the left ear, the right ear of every mouse was topically administered with 25 μL of acetone or the vehicle used for the corresponding extract (chloroform, methanol or water). In all cases, 2 mg of extract was dissolved in 25 μL of the relevant vehicle. The solvents and extracts were topically administered to both faces of each left ear treated with TPA. A control group received 2 mg/ear of indomethacin as a positive control in the ear treated with TPA.

The animals were sacrificed by cervical dislocation 6 h after the treatment. A piece of atrial tissue was then removed by drilling a 6 mm hole at the center of the ear with a punch to record its weight for further analysis. The induced edema was quantified as the weight difference between the auricular atrial tissues treated with TPA and the corresponding tissue from the other ear, where only the vehicle was applied. The inhibition of edema was defined as a percentage of the edema produced in the control animals, with the following formula:

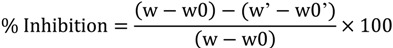
(1)
where: W = Weight of the left ear with TPA (TPA control); Wo = Weight of the right ear with vehicle (normal control); W’ = Weight of the left ear with TPA (treated); Wo’ = Weight of the right ear with vehicle (untreated).

#### 3.4.1. Differential Leukocyte Count

A sample of blood from the saphenous vein of every mouse was taken at four different times (0, 240, 300 and 360 min). Blood smears were immediately prepared on pre-cleaned microscopic slides of standard size and then treated according to Wright’s method. The time zero was collected before the TPA application. The blood smears were used for the differential leukocyte count using an Olympus CX41 microscope equipped with an Olympus U-CMAD3 photographic camera. The total number of cells counted in each of the stained slides was 100. The leukocytes were inspected using the 10× zoom, selecting the region with the best distribution of cells. The counting and sorting of leukocytes was done using the 100× immersion objective, placing it on the upper end of the blood smear and moving it toward the lower end in successive fields. When necessary, more fields were inspected until 100 cells were counted, as required by the method [70]. Neutrophils, lymphocytes, monocytes, eosinophils and basophil cells were identified and counted in the blood smears. The results were expressed as the absolute values of each studied cell type.

### 3.5. Fractionation of the Chloroform Extract

The chloroform extract was separated using a 70-230-mesh silica gel chromatographic column. The column was eluted with hexane, and the polarity was further increased with ethyl acetate. Nine fractions were collected. The first fraction was eluted only with hexane, while the second through the ninth fractions were eluted with a 98:2 hexane/ethyl acetate mixture. Fractions two and three rendered a white precipitate, which was independently evaluated. All of the fractions were stripped of solvent on a rotary evaporator under reduced pressure.

### 3.6. Purification of the Active Principles

White precipitate was filtered, dissolved in chloroform and then re-precipitated with ethyl acetate. Finally, it was filtered out, and the solid was placed in a vacuum oven to remove any trace of solvent.

### 3.7. Bioassay-Guided Chemical Study (Anti-Inflammatory Effect in the Model of Ear Edema Induced by TPA)

Evaluation of the anti-inflammatory effects of all the liquid fractions, as well as of the white precipitate obtained upon chromatographic separation of the chloroform extract, was performed according to the methodology detailed above.

### 3.8. Structural Elucidation

#### 3.8.1. Nuclear Magnetic Resonance Spectroscopy (NMR)

The NMR spectra were collected in a Bruker Avance-III 500 spectrometer, with a 5 mm ID-BB (31P-109 Ag) z- gradient field probe. Tetramethylsilane (TMS) was used as the internal reference, and CDCl_3_ and DMSO-*d*_6_ were used as solvents (Sigma-Aldrich). The ^1^H spectra were collected routinely, while the ^13^C and the 2D spectra (COSY, HSQC, HMBC) were acquired only when necessary. The assignments of the NMR spectra were performed in a systematic way.

#### 3.8.2. Infrared Spectroscopy (ATR-FTIR)

Infrared spectroscopy was performed on a micro-Raman model Lab-Ram spectrometer (HORIBA Jobin Yvon, Longjummeau, France) with an ATR lens with detection of excitation in the range of 400–4000 nm.

#### 3.8.3. High-Resolution Electrospray Ionization Mass Spectrometry

The spectra were obtained using a micrOTOF-QII mass spectrometer (Bruker Daltonics, Bremen, Germany) equipped with an electrospray ionization source (ESI). The parameters were set as follows: capillary 2700 V, nebulizer pressure 5.8 psi, dry gas flow 40 L/min, and dry gas temperature 180 °C. The sample was run in the negative ion mode. The scan range was from 50 to 3000 *m/z*.

### 3.9. Statistical Analysis

The data were analyzed by analysis of variance (ANOVA) followed by Tukey’s *post hoc* test; an alpha level of *p* ≤ 0.05 was used to determine significant differences between groups.

## 4. Conclusions

These results reveal that the chloroform extract from *Senna villosa* leaves has anti-proliferative and anti-inflammatory properties. In addition, this extract contains several compounds which are contained in the optimum amounts to be biologically active immune system stimuli, with the capacity to block activated inflammatory mechanisms at both local and systemic levels.

Three aliphatic esters, hexyl tetradecanoate (C_20_H_40_O_2_), heptyl tetradecanoate (C_21_H_42_O_2_) and octyl tetradecanoate (C_22_H_44_O_2_), were identified as the principal constituents in the white precipitate obtained from fractions 2 and 3 of the chloroform extract, and this is the first report of the presence of these compounds in *Senna villosa.*

This research provides the first evidence of the anti-proliferative and anti-inflammatory properties of extracts isolated from *Senna villosa*, suggesting that this plant may be a viable source of new drugs to inhibit inflammation of the skin in diseases such as atopic dermatitis and psoriasis. Interestingly, the chloroform extract of *Senna villosa* at the dose of 2 mg/ear exhibited better anti-inflammatory effect than indomethacin at the same dose of 2 mg/ear with a statistical significant difference. Therefore, in new studies, it is mandatory to evaluate each component independently and in different doses, looking for to propose a new anti-inflammatory drug. In addition, further investigations should provide additional biochemical data to elucidate the precise cellular mechanisms of the anti-inflammatory compounds identified, which could reveal a new therapeutic strategy for the treatment of inflammatory diseases.
